# Levodopa/carbidopa/entacapone 200/50/200 mg (Stalevo^®^ 200) in the treatment of Parkinson’s disease: a case series

**DOI:** 10.4076/1757-1626-2-7134

**Published:** 2009-07-30

**Authors:** Kapil D Sethi, Robert A Hauser, Stuart H Isaacson, Terry McClain

**Affiliations:** 1 Movement Disorders Program, Medical College of GeorgiaAugusta, Georgia 30912USA; 2 Parkinson’s Disease and Movement Disorders Center, University of South Florida4 Columbia Drive, Suite 410, Tampa, Florida 33606USA; 3 Parkinson’s Disease and Movement Disorders Center of Boca Raton951 NW 13th Street, Bldg. 5-E, Boca Raton, Florida 33486USA

## Abstract

Levodopa continues to be the most efficacious and widely used treatment for Parkinson’s disease. Levodopa dosing is understood to be critical for the optimal control of symptoms, and increasing the levodopa dose is a common method to treat advancing disease. Escalating levodopa dosages coupled with disease progression is associated with increasing likelihood of developing levodopa-induced dyskinesia. Moreover, frequent and complicated dosing schemes, combined with limited dose availability, leads to increasing pill burden and its associated impairment of patient adherence issues. Levodopa/carbidopa/entacapone has been shown to improve the pharmacokinetic profile of levodopa and provide superior symptomatic control compared with conventional levodopa/dopa decarboxylase inhibitor therapy. We report four case histories describing clinical experience of using levodopa/carbidopa/entacapone 200/50/200 mg, one of the latest doses of this formulation, in a range of patients with Parkinson’s disease. These cases illustrate that levodopa/carbidopa/entacapone 200/50/200 mg provides improvements in symptomatic control and convenience, and that switching to this dose was not associated with safety concerns.

## Introduction

After 40 years, levodopa remains the most efficacious and widely used treatment for Parkinson’s disease (PD), with the majority of patients requiring levodopa therapy at some point during the course of their disease [1-3]. However, as the disease progresses it can become increasingly difficult for patients to achieve clinical benefits, and patients will require increasingly higher doses of levodopa. In turn, this may lead to adherence-related issues as a result of the increased pill burden and complex dosing schedules [4]. Failure to manage effectively the medication regimen can contribute to functional impairment, decreased quality of life and increased motor symptoms. Therefore, maintaining patients on their therapy is a key issue for the management of PD [5].

Conventional levodopa formulations are most commonly administered with a dopa decarboxylase (DDC) inhibitor (DDCI), such as benserazide (Madopar®) or carbidopa (Sinemet®) [6,7]. In addition, controlled-release (CR) levodopa formulations were developed in an attempt to maintain a more stable plasma levodopa profile. In practice, however, these display erratic absorption, delayed ON-time and poor bioavailability, compared with immediate-release conventional levodopa [8]. In clinical practice, some physicians may use CR-levodopa/carbidopa (CR-LC; Sinemet CR®) to provide control of night-time symptoms [9]. A third formulation of levodopa has been developed, which provides dual-enzyme inhibition of DDC and catechol-*O*-methyltransferase (COMT) [10]. This formulation of levodopa/carbidopa/entacapone (LCE; Stalevo®) has been shown to extend the half-life of levodopa by up to 85% and increases its bioavailability by 35% [11,12].

Levodopa/carbidopa/entacapone has been shown to provide superior symptomatic benefits in patients with PD compared with conventional levodopa/DDCI therapy [13]. This has been shown to translate into superior treatment adherence, compared with the addition of separate entacapone tablets to levodopa/DDCI [14].

LCE is available as a single tablet in a range of dose strengths, one of the newest of which is levodopa/carbidopa/entacapone 200/50/200 mg (Stalevo® 200; LCE 200). The rationale for the development of the LCE 200 dose was to increase dose flexibility and convenience by providing a single tablet for patients requiring higher doses of levodopa who would otherwise incur an increased pill burden; to provide treatment for patients who have progressed on alternative levodopa formulations and still require additional symptom control; and to potentially provide a useful and convenient dose for night-time control and early morning akinesia. Recently, a Phase I pharmacokinetic study demonstrated that LCE 200 provides a superior PK profile to that of CR-LC 200/50 mg, when administered either as a single evening dose or as a three-times daily dosing regimen [15].

We here present case studies describing the use of LCE 200 in four patients with PD, in order to provide information on real-world experience of when and how this new dosing strength may be used in clinical practice.

## Cases presentations

We reviewed the case histories of four patients with PD (four males), treated at three clinics in the USA. The patients’ ages ranged from 48 to 72 years; PD duration ranged from 4 to 14 years (Table 1). Prior to switching to LCE 200, the patients had received levodopa therapy for between 2 and 12 years; all but one patient experienced dyskinesia and/or wearing-off prior to switching (Table 1). Further details of the individual cases are described below.

**Table 1.** Summary of case reports of four patients with Parkinson’s disease switched to LCE 200

	Case 1	Case 2	Case 3	Case 4
Sex	Male	Male	Male	Male
Age (years)	48	69	72	65
Duration of Parkinson’s disease (years)	4	14	8	13
Hoehn & Yahr stage	2	OFF 3/ON 2.5	2	2.5
Duration of levodopa therapy (years)	2	12	5	8
Motor complications	Wearing-off	Dyskinesia, wearing-off, delayed ON time, morning akinesia	None	Dyskinesia, wearing-off
Symptom benefit?	Equal	Yes	Yes	Yes
Convenience benefit?	Yes	Yes	Not stated	Not stated
AEs associated with switching to LCE 200?	No	No	No	No

AEs, adverse events; LCE 200, levodopa/carbidopa/entacapone 200/50/200 mg.

### Case report 1

A 48-year-old Caucasian male with a 4-year history of PD (Hoehn & Yahr [HY] stage 2) whose PD symptoms consisted of asymmetric tremor at rest (left more than right) and difficulty using the left arm. The patient had previously received levodopa therapy for 2 years: most recently, two LC 100/25 mg tablets five times per day, plus entacapone 200 mg five times per day (taken with each LC dose). Other PD medication consisted of rasagiline 1 mg/day; the patient had also previously received ropinirole, which caused him to feel ‘foggy minded’, and pramipexole, which was associated with compulsive gambling. The patient experienced wearing-off, characterized by a return of tremor and restlessness, sweating and anxiety. Based on both the physician’s and the patient’s assessment, treatment was switched to one LCE 200 tablet five times per day (levodopa 1000 mg/day), with the aim of improving convenience and compliance. No screening diagnostic tests were used before switching. Following the switch to LCE 200, the patient has experienced no adverse events (AEs) with LCE 200 and reports equal benefit compared with his prior therapy, but with the convenience of taking only one tablet five times daily (a total of five tablets), instead of three tablets five times daily (a total of 15 tablets).

### Case report 2

A 69-year-old Caucasian male with a 14-year history of PD (HY OFF stage 3 / ON stage 2.5), based on clinical diagnosis; whose PD symptoms consisted of right rest tremor, bradykinesia (right more than left), and rigidity. Gait was slow, and when OFF he had impaired balance. The patient also has orthostatic hypotension. The patient had previously received levodopa therapy for 12 years: most recently, one LCE 100 tablet taken with one half-tablet of LC 100/25 six times per day. Other PD medication consisted of ropinirole 8 mg/day and rasagiline 1 mg/day. The patient experienced wearing-off (with tremor, imbalance, and anxiety), morning akinesia, and delayed ON. He also had non-troublesome, peak-dose dyskinesia in the evening. As a result of the patient’s morning akinesia, delayed ON and wearing-off, the physician decided to switch the first three doses of levodopa (LCE 100 + half-tablet LC 100/25) to LCE 200, instead of increasing supplemental LC 100/25. The Unified Parkinson’s Disease Rating Scale and a comprehensive review of symptoms assessment were used for screening before switching. Following the switch of these doses to LCE 200, the patient has experienced no AEs and finds the single tablet at each dose to be more convenient. He reports less OFF time, with reduced risk of falls. His medication has a quicker onset of action in the morning. Mild evening dyskinesia persisted, but stopping the additional half-tablet dose of LC in the evening led to less dyskinesia. Subsequently, the ropinirole was switched to once-daily prolonged-release formulation, once-daily rasagiline continued, and the additional LC 100/25 with later LCE 100 doses was replaced with LCE 150. He found this medication regimen with fewer daily pills simpler to follow, and reported no increase in dyskinesia or other side effects.

### Case report 3

A 72-year-old Caucasian male with an 8-year history of PD (HY stage 2) whose PD symptoms consisted of mild right and slight left rest tremor, mildly decreased co-ordination on the right, slightly decreased coordination on the left, mildly increased tone on the right, and slightly increased tone on the left. The patient previously received levodopa therapy for 5 years: one LC 100/25 mg tablet five times per day, switched to one LCE 150 tablet four times per day and CR-LC 200/50 mg administered at bedtime. Over the course of his disease, PD medication consisted of selegiline, pergolide mesylate, amantadine and rasagiline. All were stopped due to side effects or lack of efficacy. His current medications included LCE 150 mg, four times per day and ropinirole 3 mg, three times per day. The patient experienced no motor complications on his current therapy. The patient noted no clear OFF time, but felt that his ON time was not as good as it had been. He also noticed an increased difficulty in performing activities of daily living (ADL) throughout the day, and the physician found a worsening of symptoms upon examination. As a result of these patient and physician assessments, treatment was switched to one LCE 200 tablet four times per day. No screening diagnostic tests were used before switching. Following the switch to LCE 200, the patient has experienced no AEs. He reports that his physical functioning has improved, in terms of walking, ADL and personal care. Although he noticed no change in function during the night, he notes that he is turning ON much quicker in the morning. He reports that he turns ON in about 30 minutes now where it used to take 60-90 minutes.

### Case report 4

A 65-year-old Caucasian male with a 13-year history of PD (HY stage 2.5), based on physical examination and symptoms, whose PD symptoms consisted of rest tremor on the left, moderately decreased coordination on the left, mildly decreased coordination on the right, mildly increased tone on the left and slightly increased tone on the right. The patient previously received levodopa therapy for 8 years: one LC 100/25 mg tablet three times per day, increased to five times per day; switched to one LCE 100 mg tablet six times per day, eventually increased to one LCE 150 mg tablet six times per day. Other PD medication consisted of selegiline, ropinirole, entacapone and rasagiline. The patient experienced dyskinesia for approximately 5% of the day, and wearing-off for approximately 25% of the day. He also experienced occasional dose failure. The patient experienced significant OFF time after his 07:00 and 14:00 dose, almost every day, and physical examination elicited some dyskinesias. For these reasons, the patient’s treatment was switched to LCE (a total of 1000 mg levodopa/day), administered as a combination of LCE 150 and LCE 200, divided into six doses as follows: LCE 200 at 07:00 and 14:00, LCE 150 at 05:00, 12:00, 17:00, and 21:00. The physician’s rationale for this treatment regimen was that, by only increasing the doses given at 07:00 and 14:00 (when the patient reported experiencing significant OFF time), the patient’s OFF time might improve, without increasing his dyskinesia. No screening diagnostic tests were used before switching. The patient reports a significant improvement in ON time, with no worsening of dyskinesias.

## Discussion

In this case series, all four patients experienced important benefits as a result of switching to LCE 200 mg treatment from their previous levodopa therapy: the three patients (Cases 2-4) who had progressed on high levodopa/LCE doses, experienced benefits in terms of symptom control, with two patients reporting improvements in ON time/reduction in OFF time (Cases 2 and 4), and one patient reporting improvements in physical functioning and quality of life, as determined by ADL (Case 3). For one patient (Case 2), a decrease in the OFF time may have reduced the risk of falls, and switching to doses of LCE 200 considerably simplified the patient’s dosing schedule. Interestingly, a levodopa dose reduction in the evening was useful for managing dyskinesia in this patient. The youngest patient (Case 1), who had the shortest duration of PD and previous levodopa therapy, experienced equal benefits to his prior therapy, in terms of symptom control, but reported that LCE 200 was more convenient than his previous treatment, since it involved taking one tablet five times per day, rather than three tablets five times per day. Dosing convenience and improved symptom control were the most common reason for switching to the LCE 200 dose.

All patients in this report received two to six doses of LCE 200 per day and all patients except for one (Case 3) were experiencing motor complications prior to the switch to LCE 200. Use of LCE 200 was associated with an improvement in motor complications in all three patients. Wearing-off symptoms, including the non-motor symptoms, such as anxiety and swearing, improved in all three patients. Dyskinesias were not increased in the two patients who experienced dyskinesia before the switch to LCE 200 (Cases 2 and 4). Importantly, none of the patients reported any AEs as a result of switching to LCE 200.

Switching was performed either directly from LC 100 plus entacapone (Case 1), or from LCE 150 (Cases 2-4); only one patient (Case 2) underwent diagnostic screening tests prior to switching.

LCE 200 was shown to be effective for additional control of morning akinesia (Case 2), and during the night in a patient previously treated with CR-LC 200/50 mg at bedtime in addition to LCE 150 (Case 3).

## Conclusions

These cases illustrate that LCE 200 is a useful new dose, with the potential to provide improved symptom control, greater dosing flexibility and convenience, and additional control overnight and for morning akinesia. In these cases, switching to LCE 200 was not associated with safety concerns, including increases in dyskinesia, which may be seen with increasing doses of conventional levodopa. The number of case reports presented herein is limited, since the 200 mg dose has only recently been included in clinical practice. Further clinical experience with LCE 200 will prove useful in expanding our knowledge of the potential benefits of this new dose. However, given that levodopa dosing is a key issue for the symptomatic treatment of PD, the availability of additional levodopa doses will allow physicians greater prescribing flexibility, which may enable a more individualized approach to treating patients with PD.

## Abbreviations

ADL: activity of daily living
AE: adverse events
COMT: catechol-*O*-methyltransferase
CR: controlled release
CRLC: CR-levodopa/carbidopa
DDC: dopa decarboxylase
DDCI: dopa decarboxylase inhibitor
LCE: levodopa/carbidopa/entacapone
PD: Parkinson’s disease
